# Anti-Tumor Immunogenicity of the Oncolytic Virus CF33-hNIS-antiPDL1 against Ex Vivo Peritoneal Cells from Gastric Cancer Patients

**DOI:** 10.3390/ijms241814189

**Published:** 2023-09-16

**Authors:** Zhifang Zhang, Annie Yang, Shyambabu Chaurasiya, Anthony K. Park, Sang-In Kim, Jianming Lu, Hannah Valencia, Yuman Fong, Yanghee Woo

**Affiliations:** 1 Department of Surgery, City of Hope National Medical Center, Duarte, CA 91010, USA; zhzhang@coh.org (Z.Z.); ayang@coh.org (A.Y.); schaurasiya@coh.org (S.C.); skim@coh.org (S.-I.K.); jlu@coh.org (J.L.); hvalencia@coh.org (H.V.); yfong@coh.org (Y.F.); 2 Cancer Immunotherapeutics Program, Beckman Research Institute of City of Hope National Medical Center, Duarte, CA 91010, USA; apark@coh.org

**Keywords:** malignant ascites, peritoneal washings, gastric cancer, diagnostic laparoscopy, oncolytic virotherapy, ex vivo, peritoneal immune tumor microenvironment

## Abstract

We studied the immunotherapeutic potential of CF33-hNIS-antiPDL1 oncolytic virus (OV) against gastric cancer with peritoneal metastasis (GCPM). We collected fresh malignant ascites (MA) or peritoneal washings (PW) during routine paracenteses and diagnostic laparoscopies from GC patients (n = 27). Cells were analyzed for cancer cell markers and T cells, or treated with PBS, CF33-GFP, or CF33-hNIS-antiPDL1 (MOI = 3). We analyzed infectivity, replication, cytotoxicity, CD107α upregulation of CD8^+^ and CD4^+^ T cells, CD274 (PD-L1) blockade of cancer cells by virus-encoded anti-PD-L1 scFv, and the release of growth factors and cytokines. We observed higher CD45^−^/large-size cells and lower CD8^+^ T cell percentages in MA than PW. CD45^−^/large-size cells were morphologically malignant and expressed CD274 (PD-L1), CD252 (OX40L), and EGFR. CD4^+^ and CD8^+^ T cells did not express cell surface exhaustion markers. Virus infection and replication increased cancer cell death at 15 h and 48 h. CF33-hNIS-antiPDL1 treatment produced functional anti-PD-L1 scFv, which blocked surface PD-L1 binding of live cancer cells and increased CD8^+^CD107α^+^ and CD4^+^CD107α^+^ T cell percentages while decreasing EGF, PDGF, soluble anti-PD-L1, and IL-10. CF33-OVs infect, replicate in, express functional proteins, and kill ex vivo GCPM cells with immune-activating effects. CF33-hNIS-antiPDL1 displays real potential for intraperitoneal GCPM therapy.

## 1. Introduction

Peritoneal metastases (PM) cause therapeutic failure in gastric cancer (GC) patients and indicate a dismal prognosis [1,2,3,4]. Synchronous peritoneal involvement in GC can be detected in up to 21% of patients [2]. Gastric cancer with peritoneal metastases (GCPM) is treatment-resistant and progresses rapidly through systemic and regional therapeutic interventions [2,3,5,6]. Therapeutic failure in the peritoneum is experienced within a few months of starting treatment, with a median overall survival of less than eight months [7]. Treatment responses and peritoneal disease progression are predominantly governed by genomically stable gastric tumors, the severely immunosuppressive peritoneal tumor microenvironment (TME), and the blood-peritoneal barrier that limit systemic drug delivery to peritoneal tumors [8,9].

Oncolytic viral therapy presents a promising platform against GCPM. Naturally occurring and genetically modified oncolytic viruses (OVs) selectively replicate in cancer cells by hijacking the dysregulated cancer cell genome to cause lysis. Early trials have established the safety of genetically modified OVs, including those derived from adenovirus, Herpes simplex virus-1 (HSV-1), and poxviruses [10,11,12,13]. OV-mediated cell death is immunogenic due to the release of viral pathogen-associated molecular patterns (PAMPs) and cell-derived damage-associated molecular patterns (DAMPs). Furthermore, the OV-mediated lysis of cancer cells facilitates the release of tumor-associated antigens (TAAs). Together, these features initiate pro-immunogenic signaling to promote type I interferon and cytokine production, amplify the immune response, and further enhance anti-tumor activity through antigen immune memory [14,15,16,17,18,19]. 

We are investigating a chimeric orthopoxvirus, CF33, and its derivatives armed with transgenes to deliver clinically relevant proteins, namely human sodium iodide symporter (hNIS) and anti-PD-L1 inserted as a single-chain variable fragment (scFv). CF33-hNIS (VAXINIA) and CF33-hNIS-antiPDL1 (CHECKVacc) have demonstrated preclinical safety and efficacy in a broad spectrum of solid tumors and entered phase I clinical trials as intratumoral (IT) and intravenous (IV) therapies against triple-negative breast cancer (TNBC) (NCT05081492) and metastatic solid tumors (NCT05346484), respectively. We have previously shown that CF33-OVs upregulate PD-L1 in vitro and in vivo in pancreatic cancer and breast cancer [20,21]. Intraperitoneal (IP) CF33-hNIS-antiPDL1 delivery led to robust peritoneal tumor oncolysis with decreased MA and improved survival in a mouse GC model [22] and pancreatic cancer model [23]. Here, we investigated the ex vivo anti-tumor activity of CF33-hNIS-antiPDL1 and its effects on peritoneal-associated cell subsets using GCPM patient samples. We demonstrate a strong oncolytic and immune-activating effect of CF33-hNIS-antiPDL1 against GCPM ex vivo.

## 2. Results

### 2.1. Baseline Characteristics of Patients

The baseline demographic and clinical characteristics of 27 patients are shown in Table 1. There were 14 males (51.8%) and 13 females (48.2%) with a mean age of 53.5  ±  15.5 years. The study cohort was ethnically heterogeneous and included 8 Asian (29.6%), 10 Hispanic (37%), 8 Non-Hispanic White (NHW, 29.6%), and 1 Black person (3.7%). Eighteen patients (66.7%) had confirmed histologic evidence of peritoneal carcinomatosis/malignant cells, and 4 of these 18 patients also presented with other sites of distant metastases (liver or ovaries). Primary gastric tumors were characterized by 88.9% poorly differentiated adenocarcinoma (n = 24 versus n = 2 well-differentiated and n = 1 moderately differentiated), 92.6% advanced T stage (n = 25 T4 versus n = 1 T3 and n = 1 T2), 77.8% diffuse-type (n = 21) and 22.2% intestinal-type (n = 6) Lauren classifications. Of the peritoneal biospecimens collected, 14 were MA (51.9%), 4 were PW(+) (14.8%), and 9 were PW(−) (33.3%).

### 2.2. PW(+) and MA Groups Show a Lower CD8^+^ T Cell Percentage

**Figure 1:**
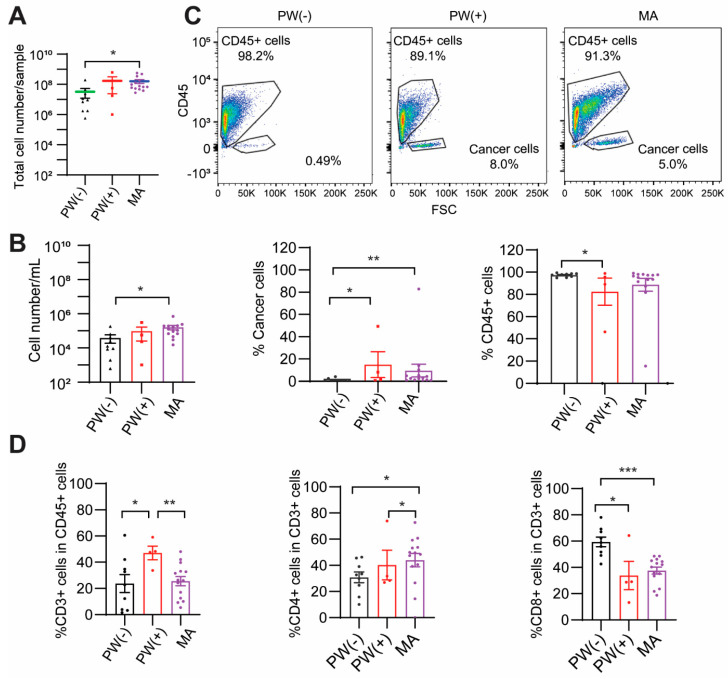
**Cancer cell percentage and T cell subsets in ex vivo cells from GCPM patients.** Patients were divided into three groups: peritoneal washing (PW(−)), peritoneal washing with malignant metastasis using cytology (PW(+), early stage metastasis), and malignant ascites (MA, late-stage metastasis). (**A**). Total cell numbers. (**B**). Cell number/mL. (**C**). Percentage of leukocytes and cancer cells. CD45^+^ cells are leukocytes, and CD45^−^/large-size cells were considered cancer cells. (**D**). Percentage of CD3^+^, CD3^+^CD4^+^, and CD3^+^CD8^+^ T cells. Mean ± SEM. * *p* < 0.05, ** *p* < 0.01, *** *p* < 0.001. Black: PW(−), Red: PW(+), Purple: MA. PW(−): peritoneal washing (cytology negative); PW(+): peritoneal washing (cytology positive); MA: malignant ascites.

Our results showed that the MA group had a higher number of total cells (*p* < 0.05) and a higher number of cells per mL of washing or ascites volume (*p* = 0.05) than the PW(−) group (Figure 1A,B). Cells from fresh PW(−), PW(+), and MA were stained with lineage markers of leukocytes (CD45) and T cells (CD3, CD4, CD8) and analyzed using flow cytometric analysis. We initially selected CEA as a GC cell marker. However, staining results showed that CEA was weakly expressed on the peritoneal cancer cell surface and hence could not be used to separate cancer cells from other cell types. Thus, the leukocyte marker CD45 and cell size (forward scatter, FSC) were used to separate CD45^+^ leukocytes and CD45^−^/large-size cancer cells. CD45^−^/large-size cells were morphologically malignant. Our results showed a significantly higher percentage of cancer cells in the PW(+) and MA groups compared to the PW(−) group (*p* < 0.05 or *p* < 0.01, respectively). A significantly lower percentage of CD45^+^ leukocytes was seen in the PW(+) group (*p* < 0.05, Figure 1C). It is noted that 0.49% of CD45^−^/large-size cells in the cytology-negative PW(−) group may represent the normal resident non-immune cells in the peritoneal cavity (Figure 1C). However, the PW(+) and MA groups had a ten times higher percentage of CD45^−^/large-size cells than the PW(−) group, so these CD45^−^/large-size cells most likely represent the cancer cell population in PW(+) and MA groups. While no significant differences were observed in CD3^+^ T cell percentage between MA and PW(−) groups, a significantly higher percentage of CD4^+^ T cells (*p* < 0.05) and lower of CD8^+^ T cells (*p* < 0.001) were seen in the MA group than the PW(−) (Figure 1D). Interestingly, in the PW(+) group, which is clinically considered to be an earlier stage of peritoneal metastasis, the percentage of CD3^+^ T cells was significantly higher than in both the PW(−) and MA groups (*p* = 0.05 or *p* < 0.01, respectively). The proportion of CD8^+^ T cells in the PW(+) was similar to the MA group and significantly lower than the PW(−) group (*p* < 0.05). Our results indicate that GC patients with peritoneal cancer cells have a relatively immunosuppressive peritoneal cellular make-up reflected in fewer CD8^+^ T cells than in the PW(−) group, which did not have peritoneal disease. 

### 2.3. GCPM Cells Express PD-L1, CD252, and EGFR

**Figure 2:**
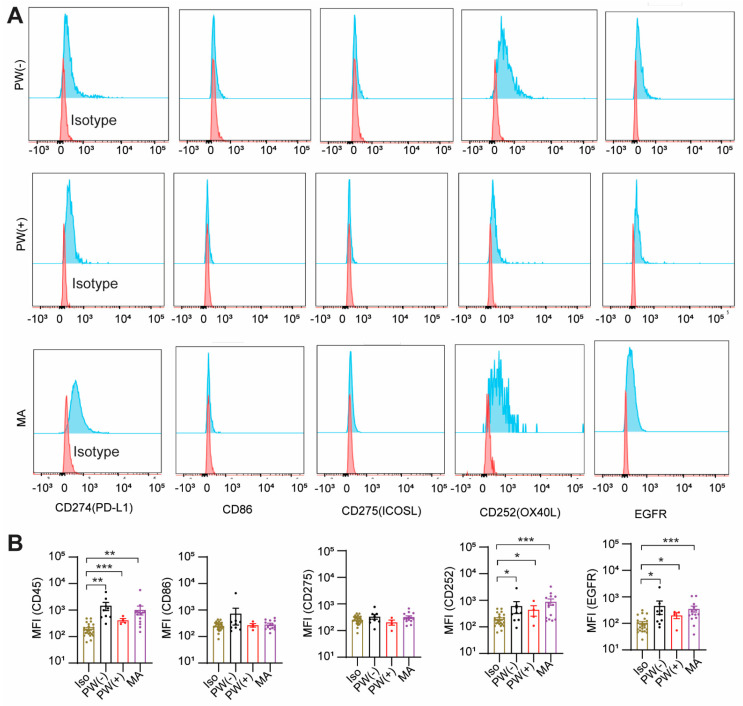
**Surface marker expressions of ex vivo GCPM cancer.** Fresh ex vivo cells were stained with anti-CD45 antibody and the markers shown in the figure. CD45^−^/large-size cells were considered cancer cells. (**A**) One representative of each marker. (**B**) Statistical analysis (Mean ± SEM). * *p* < 0.05, ** *p* < 0.01, *** *p* < 0.001. Iso: Isotype; PW(−): peritoneal washing (cytology negative); PW(+): peritoneal washing (cytology positive); MA: malignant ascites. Brown: Isotype; Black: PW(−); Red: PW(+); Purple: MA.

We observed that the immune checkpoint inhibitor, PD-L1 (CD274), a ligand for T cell surface receptor PD-1, was expressed on the cancer cell surface of PW(+) and MA groups similar to the PW(−) group, compared to isotype control (*p* < 0.001, *p* < 0.01, or *p* < 0.01, respectively). There was no significant difference in PD-L1 expression between the PW(−) and MA groups. CD86, a ligand of T cell surface receptor CTLA4, was not expressed on cancer cells (Figure 2A,B). Interestingly, CD252 (OX40L), the ligand of OX40, a T cell activation marker, was expressed on cancer cells of the PW(+) and MA groups similar to the PW(−) group (*p* < 0.05, *p* < 0.001, *p* < 0.05, respectively). CD275 (ICOSL), the ligand of ICOS, also a T cell activation marker, was not expressed on cancer cells (Figure 2A,B). The epithelial growth factor receptor (EGFR) was significantly higher expressed on cancer cells of the PW(+) and MA groups similar to the PW(−) group (*p* < 0.05, *p* < 0.001, *p* < 0.05, respectively, Figure 2A,B) with no significant difference among the three groups. These results suggest that GC cells in the peritoneum are positive for CD274, CD252, and EGFR.

Since a lower CD8^+^ T cells percentage and higher CD4^+^ T cells percentage were shown in the MA group compared to PW(−) group, we examined the activation and exhaustion markers on CD4^+^ and CD8^+^ T cells using flow cytometry. While the CD8^+^ T cells were present in all groups, they were negative for both the activation markers CD25 and CD107α, and exhaustion markers, including CTLA4, LAG3, CD160, and BTLA (Supplemental Figure S1A). CD4^+^ T cells expressed low levels of CD107α but were negative for other markers (Supplemental Figure S1B). Both CD4^+^ and CD8^+^ T cells stained negative for PD1. These results suggest that CD8^+^ and CD4^+^ T cells in PW(−) and MA are in a non-activated state.

### 2.4. CF33-hNIS-antiPDL1 Kills GCPM Cells Ex Vivo

**Figure 3:**
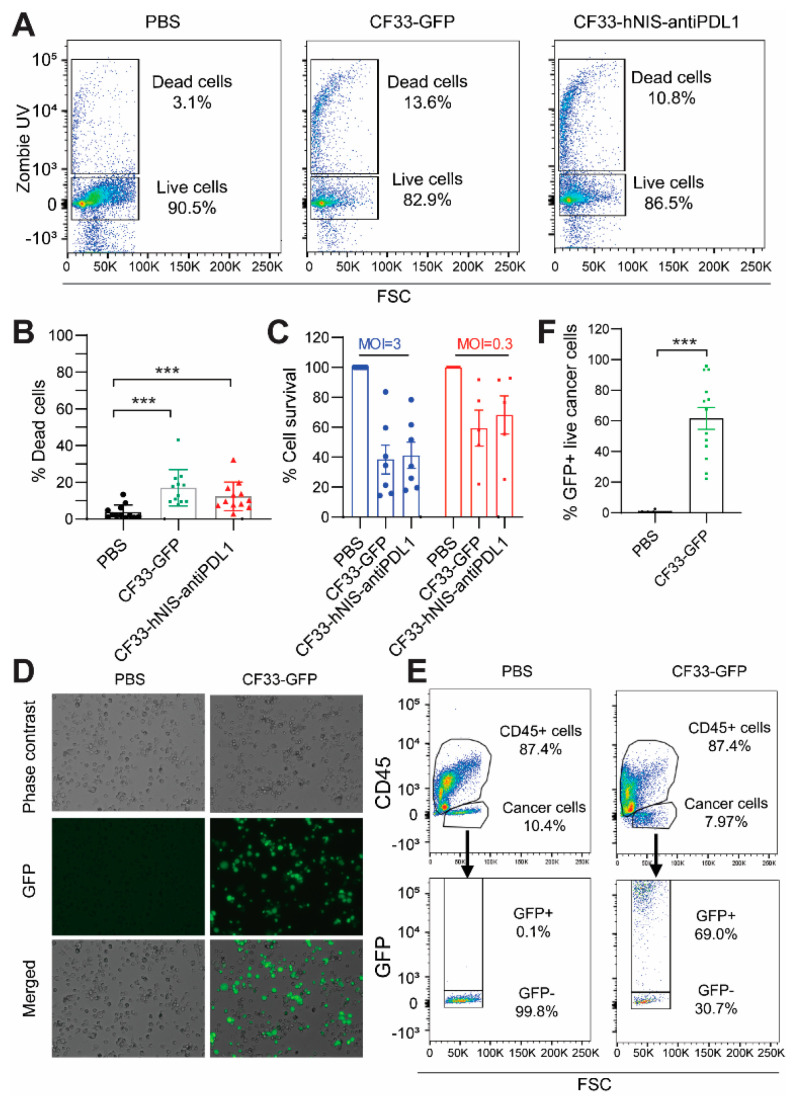
**Cytotoxicity and infection of ex vivo cells treated with CF33-GFP or CF33-hNIS-antiPDL1**. (**A**,**B**). Ex vivo cells (2.5 × 10^6^/mL) were treated with PBS, CF33-GFP (MOI = 3), or CF33-hNIS-antiPDL1 (MOI = 3) for 15 h, harvested, stained with Zombie UV for dead cells, and analyzed using flow cytometry for (**A**) representatives and (**B**) statistical analysis. (**C**) Ex vivo cells (1 × 10^5^ cells/well in 96-well plate) were treated with PBS, CF33-GFP, or CF33-hNIS-antiPDL1 for 48 h, and examined for cytotoxicity. Percent survival was calculated relative to PBS-infected wells. (**D**–**F**). Ex vivo cells (2.5 × 10^6^ cells/mL) were treated with PBS or CF33-GFP (MOI = 3) for 15 h, then imaged using fluorescence microscopy to observe CF33-GFP replication (**D**) and harvested and analyzed using flow cytometry for GFP expression of live cancer cells (**E**), representatives with statistical analysis (**F**) (Mean ± SEM). *** *p* <0.001. MOI: multiplicity of infection; GFP: green fluorescent protein.

Since there were not enough cells in the PW(−) group for virus treatment, we selected 12 samples with high cell numbers (10 from the MA group and 2 from the PW(+) group) for the OV studies. Our results showed a statistically greater percentage of dead cells at 15 h after treatment with CF33-GFP (13.6%) or CF33-hNIS-antiPDL1 (10.8%) at an MOI of 3 than with PBS (3.1%) (*p* < 0.001, respectively, Figure 3A,B). The cytotoxicity assay also showed that both CF33-GFP and CF33-hNIS-antiPDL1 killed cancer cells in a dose-dependent manner at 48 h (Figure 3C). Fluorescence microscopy confirmed GFP expression in the CF33-GFP-treated group (Figure 3D). To distinguish the types of cells infected by the virus, we stained CF33-GFP-treated ex vivo cells with lineage markers for flow cytometric analysis. After gating live cancer cells, 69% were positive for GFP at the 15 h time point (*p* < 0.001, Figure 3E,F). We also compared the viral efficacy in ex vivo cancer cells from patients receiving systemic treatment to those who were systemic therapy-naive. No difference in cancer cell killing by CF33-GFP and CF33-hNIS-antiPDL1 between these two groups was observed (Supplemental Figure S2). Our results demonstrate that CF33-hNIS-antiPDL1 and CF33-GFP infect, replicate in, and kill the ex vivo cancer cells of GCPM patients.

### 2.5. CF33-hNIS-antiPDL1 Expresses Functional anti-PD-L1 scFv in Ex Vivo Cancer Cells

**Figure 4:**
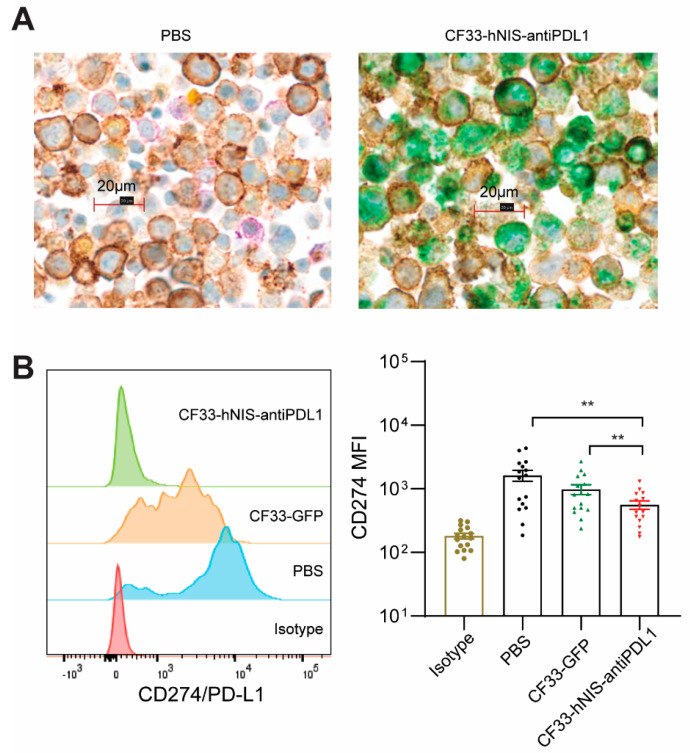
CF33-hNIS-antiPDL1-encoded anti-PD-L1 scFv was expressed in ex vivo cells and blocked PD-L1 binding of live ex vivo cancer cells. (**A**) Ex vivo cells (5 × 10^6^ cells/mL) were treated with PBS or CF33-hNIS-antiPDL1 (MOI = 3) for 15 h, then harvested and stained using multiplex immunohistochemistry for EpCAM (brown by anti-EpCAM antibody for cancer cells), PD-L1 (purple by anti-PD-L1 antibody), virus expressed anti-PD-L1 scFv protein (green by anti-Flag Tag/DDDDK antibody), and Treg cells (yellow by anti-Foxp3 antibody). (**B**) Ex vivo cells (2.5 × 10^6^ cells/mL) were treated with PBS, CF33-GFP (MOI = 3), or CF33-hNIS-antiPDL1 (MOI = 3) for 15 h, then harvested, stained with PE-anti-human CD274 (PD-L1) antibody, and analyzed using flow cytometry shown as one representative of CD274 expression (left, histogram) with statistical analysis of mean fluorescence intensity (MFI) (right, Mean ± SEM). ** *p* < 0.01.

The anti-PD-L1 scFv produced from CF33-hNIS-antiPDL1-infected cancer cells has the potential to block PD-L1, preventing the PD-L1-PD1-mediated exhaustion of CD8^+^ T cells. We used mIHC to visually analyze GCPM cells at 15 h after treatment with PBS or CF33-hNIS-antiPDL1 for anti-PD-L1 scFv (green), EpCAM (brown for cancer cells), PD-L1 (purple), and Treg cells (yellow for FoxP3) expression. mIHC enabled an automated, tissue-sparing, and cost-effective solution for the multiplex analysis of multiple markers on a single FFPE slide. Our results show that anti-PD-L1 scFv (green) was expressed by CF33-hNIS-antiPDL1-infected ex vivo GCPM cells, and colocalized with EpCAM-positive cancer cells. PD-L1 (purple) was significantly blocked in the CF33-hNIS-antiPDL1-treated cells (Figure 4A, right) compared to the PBS-treated cells (Figure 4A, left). We also performed flow cytometric analysis to verify the ability of secreted anti-PD-L1 scFv to block PD-L1 on cancer cells. Following treatment with CF33-hNIS-antiPDL1 or CF33-GFP (both MOI = 3) for 15 h, cells were harvested and stained with a PE-conjugated anti-PD-L1 antibody. Our results showed that PE-conjugated anti-PD-L1 binding to PD-L1 on live cancer cells was significantly blocked in the CF33-hNIS-antiPDL1-treated cells compared with both PBS control and CF33-GFP (*p* < 0.01, respectively, Figure 4B). These results suggest that CF33-hNIS-antiPDL1 infects, replicates in ex vivo GCPM cells, and produces anti-PD-L1 scFv in ex vivo culture.

### 2.6. CF33-hNIS-antiPDL1 Increases T Cell Activation and Decreases Growth Factor Release in Ex Vivo GCPM Cells

**Figure 5:**
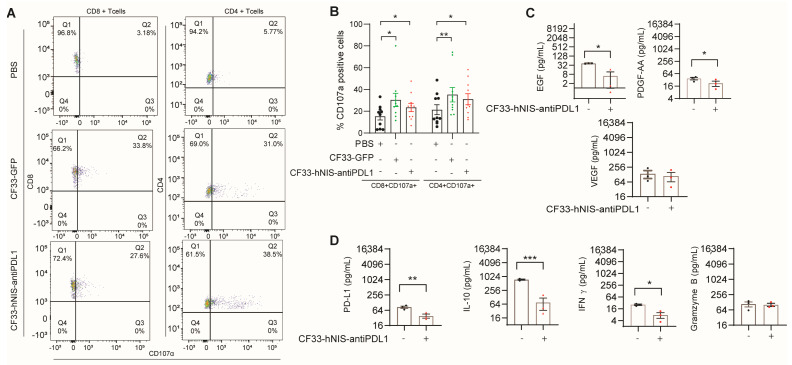
**CF33-hNIS-antiPDL1 induced CD107α expression in ex vivo CD4^+^ and CD8^+^ T cells and decreased growth factor release.** Ex vivo cells (2.5 × 10^6^ cells/mL) were treated with PBS, CF33-GFP (MOI = 3), or CF33-hNIS-antiPDL1 (MOI = 3) for 15 h, then harvested, stained with antibodies, and analyzed using flow cytometry. (**A**) One representative to show CD107α expression as dot blot analysis. (**B**) Statistical analysis of CD8^+^CD107α^+^ T cell and CD4^+^CD107α^+^ T cell percentage. (**C**,**D**) Ex vivo cells (2.5 × 10^6^ cells/mL) were treated with PBS or CF33-hNIS-antiPDL1 (MOI = 3) for 15 h, then supernatants were harvested and analyzed using Cytokine Multiplex Analysis Kit for growth factors (**C**) and soluble PD-L1/cytokines (**D**) (unit: pg/mL, n = 3, Mean ± SEM). * *p* < 0.05, ** *p* < 0.01, *** *p* < 0.001.

To analyze the effect of the virus treatment on the functionality of T cells in the ex vivo TME, we assessed T cell activation and exhaustion markers using flow cytometric analysis. A significantly higher percentage of CD107α^+^ CD8^+^ T cells was seen in virus-treated samples (33% in CF33-GFP and 27% in CF33-hNIS-antiPDL1) compared to the PBS-treated samples (3%) (Figure 5A,B). Likewise, a significantly higher percentage of CD107α^+^ CD4^+^ T cells was seen in virus-treated samples (31% in CF33-GFP and 38% in CF33-hNIS-antiPDL1) compared to the PBS-treated samples (5%) (Figure 5A,B). However, we did not observe a change in the levels of the activation marker CD25 (IL-2 receptor) or exhaustion markers CTLA4, LAG3, CD160, and BTLA on CD8^+^ and CD4^+^ T cells. Thus, our results suggest that CF33-GFP and CF33-hNIS-antiPDL1 uniquely increase the activation marker CD107α on both CD8^+^ and CD4^+^ T cells in ex vivo GCPM.

We also performed cytokine multiplex analysis to study cytokine and growth factor changes in the supernatant of GCPM cells treated for 15 h with CF33-hNIS-antiPDL1. We observed a significant decrease in tumor growth factors, such as epithelial growth factor (EGF) and platelet-derived growth factor (PDGF-AA) (*p* < 0.05, respectively, Figure 5C). Soluble PD-L1 (checkpoint protein, *p* < 0.01), IL-10 (immune inhibitor, *p* < 0.001), and IFN-γ (*p* < 0.05) significantly decreased, but granzyme B produced from CD8^+^ T cells did not change following treatment with CF33-hNIS-antiPDL1 (Figure 5D). Our results suggest that CF33-hNIS-antiPDL1-treated GCPM cells release fewer tumor growth factors and immune inhibitors in the ex vivo GCPM TME.

## 3. Discussion

The accumulation of MA within the peritoneal cavity represents a severely immunosuppressive TME dominated by M2 macrophages, and a low percentage of cytotoxic T cells [24,25,26]. The dynamic interaction between the immune cells, cancer cells, and various cytokines and growth factors can promote peritoneal disease progression in patients with MA. In this ex vivo study, we demonstrate CF33-OV’s ability to kill fresh peritoneal cancer cells obtained from MA and PW(+) from GC patients. At the 15 h time point, 69% of live cancer cells were positive for virus-encoded GFP, indicating efficient virus infection and replication in these cells. Furthermore, our study discovered that PD-L1, but not CTLA4, is expressed on the cancer cell surface and anti-PD-L1 scFv from CF33-hNIS-antiPDL1-infected cells significantly blocked PD-L1 on cancer cells. This finding is consistent with our published results in human pancreatic [20] and human GC cell lines [22]. In the ex vivo environment, CF33-hNIS-antiPDL1 also significantly increased the percentage of activated CD4^+^ and CD8^+^ T cells (CD107α^+^) compared to the PBS treatment. There was no difference in the expression of T cell markers between the CF33-hNIS-antiPDL1 and CF33-GFP treatment despite PD-L1 blocking on cancer cells by the virus-produced anti-PD-L1 scFv antibody. This could be because CD8^+^ and CD4^+^ T cells in the ex vivo peritoneal specimens were negative for PD1 and CD160, CTLA4, BTLA, and LAG3 expression. Furthermore, CF33-hNIS-antiPDL1 significantly decreased several growth factors, such as EGF and PDGF, and immune inhibitory regulators, such as soluble PD-L1 and IL-10. However, granzyme B levels, released by effector immune cells, were unaltered by virus infection. These data suggest that virus treatment could induce an anti-tumor immune TME. 

Notably, CF33-hNIS-antiPDL1 decreased IFN-γ release in ex vivo conditions. IFN-γ is a cytotoxic cytokine, which, together with granzyme B and perforin, initiates tumor cell apoptosis and enables the synthesis of immune checkpoint molecules such as PD-L1 and indoleamine-2,3-dioxygenase (IDO) to stimulate immune-suppressive mechanisms [27] Other studies have reported that IFN-γ production is regulated by natural killer (NK) and NK-T cells as part of the innate immunity, and during adaptive immune responses, CD8^+^ and CD4^+^ T cells are primary paracrine sources of IFN-γ [28]. The reason for and mechanism of IFN-γ decrease need to be further explored in the ex vivo setting.

The CF33-OV genome is a mixture of several strains of orthopoxviruses, including the vaccinia virus. Other large double-stranded DNA viruses, such as GL-ONC1, Pexa-Vec, vvDD, and MVA-FCU1, have shown excellent safety profiles in phase I clinical trials [29,30,31,32,33,34,35,36]. Recently, IT CF33-hNIS-antiPDL1 (CHEKvacc) treatment of TNBC has been safely administered to the first dosing cohort and moved to the second dosing cohort. The insights about the direct oncolytic and immune-associated effects of CF33-OVs in the peritoneal cells of GC patients will help guide the translation of CF33-OVs into early phase trials in the IP treatment of GCPM. 

In summary, routinely available peritoneal liquid biopsy specimens from GC patients provided a simple ex vivo platform for investigating the efficacy of the CF33-hNIS-antiPDL1 treatment. We show that: (1) CF33-hNIS-antiPDL1 effectively infects and kills human peritoneal GCPM cancer cells, (2) anti-PD-L1 scFv expressed by the virus-infected cancer cells blocks PD-L1 on cancer cells, and (3) CF33-hNIS-antiPDL1 decreases immune inhibitory factors and growth factors to create an anti-tumor immune microenvironment. These findings encourage the further development of CF33-hNIS-antiPDL1 into effective immunotherapeutic agents for the IP treatment of GCPM patients. 

## 4. Materials and Methods

### 4.1. Clinical Samples

We collected peritoneal fluid either as malignant ascites (MA) or peritoneal washings (PW) between January 2019 and December 2020 from 27 patients divided into three cohorts—(A) PW(−)-cytology negative; (B) PW(+)-cytology positive; and (C) MA-cytology positive, based on clinical presentation and cytologic evaluation. The study was approved by the City of Hope Institutional Review Board (IRB) of the Human Research Ethics Committee (HREC), and written informed consent was obtained from participants (IRB#18209 and IRB#19127). Patients with primary tumor biopsy-confirmed diagnosis of gastric adenocarcinoma with or without histologic confirmation of PM were enrolled regardless of prior treatment. The specimens were collected either as MA (up to 5 L at the time of diagnostic or therapeutic paracentesis) or as PW (<1 L) after peritoneal lavage with 250 cc of normal saline of the four quadrants of the peritoneum: right upper quadrant, left upper quadrant, right lower quadrant, and left lower quadrant at the time of staging laparoscopy. Immediately after peritoneal fluid removal, the specimens were divided, placed on wet ice, and transported to the clinical cytology core for cytological evaluation by a City of Hope pathologist (60–120 cc) and to the research laboratory (the remaining volume). 

### 4.2. Ascites Fractionation

In the research laboratory, the remaining fluid was gently inverted to create an even suspension and passed through a 70 μm cell strainer into a 50 mL tube. The filtrate was deemed to contain single cells, which were centrifuged for 5 min at 300× *g* to pellet the cells. Visible erythrocytes in the pellet were removed using lysing buffer. Single cells were counted and analyzed using flow cytometry for immune markers of cancer cells and T cells, or maintained in RPMI 1640 medium supplemented with 10% fetal bovine serum (FBS) and 1% antibiotic-antimycotic solution (AAS) for further treatments [37].

### 4.3. Antibodies, Reagents, CF33-GFP and CF33-hNIS-antiPDL1 Viruses

Supplementary Table S1 lists information for antibodies and reagents. CF33-GFP and CF33-hNIS-antiPDL1 [23] were generated in our lab as previously described. Briefly, CF33 is the chimeric orthopoxvirus without genetic modification. CF33-GFP has a *GFP* cassette in the *J2R* locus. CF33-hNIS-antiPDL1 has the *hNIS* cassette inserted in the *J2R* locus with *single-chain anti-PD-L1 cDNA* inserted into the *F14.5L* gene under vaccinia H5 early promoter control [22].

### 4.4. Cytotoxicity Assay

Cells were seeded at 1 × 10^5^ cells/well into 96-well plates with 100 μL/well of RPMI1640 with 10% FBS plus 1% AAS and incubated overnight. Viral constructs were thawed on ice and sonicated for 1 min. CF33-GFP and CF33-hNIS-antiPDL1 were used at multiplicity of infection (MOI) of 3 and 0.3 in RPMI1640 with 2.5% FBS and 1% AAS. Cell survival relative to PBS-infected cells was measured in duplicate at 48 h via MTS cell proliferation assay using CellTiter 96 Aqueous One solution (Promega, Madison, WI, USA) on a spectrophotometer (Tecan Spark 10M) at 490 nm [23].

### 4.5. Cytokine Multiplex Analysis

Fresh ex vivo cells from MA were plated at 2.5 × 10^6^ cells/mL in RPMI1640 containing 10% FBS and 1% AAS in 6-well plates and incubated with PBS or CF33-hNIS-antiPDL1 (MOI = 3) for 15 h. The supernatant was harvested and analyzed using a Human Magnetic XL Cytokine Discovery Panel (R&D systems, Catalog number# LKTM014) to determine cytokine concentrations according to the manufacturer’s instructions.

### 4.6. Flow Cytometric Analysis

Fresh ex vivo cells from MA or PW were blocked with 10% human serum and stained with specific antibodies, and if cell numbers were large enough, cells were plated at 2.5 × 10^6^ cells/mL in RPMI1640 containing 10% FBS plus 1% AAS in 6-well plates and incubated with PBS, CF33-GFP (MOI = 3), or CF33-hNIS-antiPDL1 (MOI = 3) for 15 h. Cells were harvested, washed with PBS, blocked with 10% human serum in PBS for 15 min on ice, stained with lineage markers and isotype controls or specific antibodies, and washed thrice with 1% BSA PBS. Cells were then fixed with 4% paraformaldehyde for 20 min and assessed using a BD LSRFortessa Flow Cytometer (BD Biosciences, San Jose, CA, USA). All results were analyzed using FlowJo software (FlowJo v10.7.2, FlowJo LLC, Ashland, Oregon) and shown as histograms, mean fluorescence intensity (MFI), or cell percentage [20].

### 4.7. Fluorescence Microscopy

Ex vivo MA cells were treated with CF33-GFP (MOI = 3) or PBS for 15 h, and images were acquired using an EVOS^®^ FL Auto Cell Imaging System (Life Technologies Corporation, Carlsbad, CA, USA). All images were adjusted identically.

### 4.8. Multiplex Immunohistochemistry (mIHC)

Ex vivo MA cells were treated with CF33-hNIS-antiPDL1 (MOI = 3) or PBS for 15 h. Cells were harvested, fixed with 10% formalin, and archived as formalin-fixed paraffin-embedded (FFPE) blocks. Tissue blocks were sectioned at 4 μm and put on positively charged glass slides (Fisherbrand (Waltham, MA, USA) Superfrost Plus Microscope Slides, precleaned). The sections were baked for 3 h at 58–60 °C before IHC staining to avoid tissue falling off. mIHC was performed on a Ventana Discovery Ultra automated stainer (Roche Diagnostics, Indianapolis, IN, USA). Sections were de-paraffinized, rehydrated, and treated by endogenous peroxidase activity inhibition and antigen retrieval with CC1 buffer (Cell conditioning 1; PH 8.5, Roche Diagnostics, Indianapolis, IN, USA). Each antigen was sequentially detected, and heat inactivation (95° and 100 °C) was performed to prevent antibody cross-reactivity between the same species. The following is the primary antibody information and staining order: EpCAM-DAB/PD-L1-Purple/FOXP3-Yellow/DDDDK-Green. Following each primary antibody incubation, DISCOVERY anti-Rabbit HQ or NP or DISCOVERY anti-Mouse HQ or NP were incubated and followed by DISCOVERY anti-HQ-HRP or anti-NP-AP. The stains were visualized using a DISCOVERY ChromoMap DAB kit (Brown), DISCOVERY Purple Kit, Yellow Kit, and Green kit (Roche Diagnostics, Indianapolis, IN, USA) accordingly, and counterstained with hematoxylin (Ventana) and coverslipped. 

### 4.9. Statistical Analysis

Assay results are expressed as mean ± SEM. Paired or unpaired Student’s *t*-tests were used for comparisons. Group comparisons for continuous data were conducted using one-way ANOVA. *p* < 0.05 was considered significant. Data were analyzed using GraphPad Prism software (version 8, GraphPad Software, San Diego, CA, USA). 

## Figures and Tables

**Table 1:** Clinicopathological features of GCPM patients.

	Number of Patients	Percentage (%)
**Total patients**	27	100
**Age (year)**		
20–39	7	25.9
40–59	10	37
60+	10	37
**Gender**		
Male	14	51.8
Female	13	48.2
**Extent of metastatic disease**		
No PM	9	33.3
PM only	14	51.8
PM plus	4	14.8
**Race/ethnicity**		
Asian	8	29.6
Hispanic	10	37
Non-Hispanic White	8	29.6
Black	1	3.7
**Clinical T**		
T1	0	0
T2	1	3.7
T3	1	3.7
T4	25	92.6
**M-stage**		
M0	2	7.4
M1	25	92.6
**Histology type**		
Intestinal	6	22.2
Diffuse	21	77.8
**PET/CT**		
positive	22	81.5
negative	5	18.5
**Grade**		
Well-differentiated	2	7.4
Moderately differentiated	1	3.7
Poorly differentiated	24	88.9
**Peritoneal cytology**		
PW(−)	9	33.3
PW(+)	4	14.8
MA	14	51.9

PM: peritoneal metastases; PET/CT: positron emission tomography/computed tomography; PW: peritoneal washings; MA: malignant ascites.

## Data Availability

All data have been presented in the manuscript.

## Supplementary Materials

The following supporting information can be downloaded at: https://www.mdpi.com/article/10.3390/ijms241814189/s1.
