# Supplementary material for: Anti-Tumor Immunogenicity of the Oncolytic Virus CF33-hNIS-antiPDL1 against Ex Vivo Peritoneal Cells from Gastric Cancer Patients
Source: Int J Mol Sci. 2023 Sep 16;24(18):14189. doi: 10.3390/ijms241814189 (PMC10532045; doi:10.3390/ijms241814189)
Supplement: Supplementary file 1 [file ijms-24-14189-s001.zip › ijms-2601837-supplementary.pdf]

SUPPLEMENTAL MATERIAL:

Supplemental Table S1. Antibody and reagent information.

Reagents Name	company	Catalog #	Clone #	Lot #	Expire
Anti-h CD45-BUV395	BD	563792	HI30	9269807	
BD Pharm Lyse™ lysing solution	BD	555899		8149712	
Anti-h CD4-BV510	Biolegend	317444	OKT4	B292551	
Anti-h CD8a-PE-Cy5	Biolegend	300910	HIT8a	B300799	
Anti-h CD14-Alexa 700	Biolegend	325614	HCD14	B243612	
Anti-h CD3-APC-Cy7	Biolegend	317342	OKT3	B282905	
Anti-h CD274-PE	Biolegend	329706	29E.2A3	B272166	
Mouse isotype PerCP/Cy5.5	Biolegend	400338	MPC-11	B275227	
Mouse isotype PE	Biolegend	402204	27-35	B271045	
Mouse isotype APC	Biolegend	402206	27-35	B272605	
Anti-h CD86-APC	Biolegend	374208	BU63	B262802	
Anti-h CTLA4-PerCP/Cy5.5	Biolegend	369608	BNI3	B267053	
Anti-h CD107α-PE	Biolegend	328608	H4A3	B283846	
Anti-h CD160-PerCP/Cy5.5	Biolegend	341210	BY55	B262314	
Anti-h CD223-PE(LAG-3)	Biolegend	369206	7H2C65	B271246	
Anti-h CD25-APC	Biolegend	302610	BC96	B258747	
Anti-h CD80-FITC	Biolegend	305206	2D10	B219028	
Anti-h CD279-PerCP/Cy5.5 (PD1)	Biolegend	329914	EH12.2H7	B267952	
Zombie UV dye	Biolegend	423108			
Anti-h CEA-Alexa 488	R&D Systems	FAB41281G	487609	1536943	
Human XL Cytokine Luminex Performance Assay Fixed Panel	R&D Systems	LKTM014		P189369	
Pooled normal human male AB Serum	Innovative Research	IPLA-SERAB-27146			Jul2023
Anti-human EPCAM:	Ventana	760-4383	1Ber-EP4	V0002926	
Anti-human PD-L1	Ventana	740-4907	SP263	F31218	
Anti-human Foxp3	abcam	ab20034	236A/E7	GR3409148-8	
Anti-FLAG Tag (DYKDDDDK)	Cell Signaling	14793	D6W5B	No.4	
Paraformaldehyde 32%	Electron Microscopy Sciences	15714		181210-10	

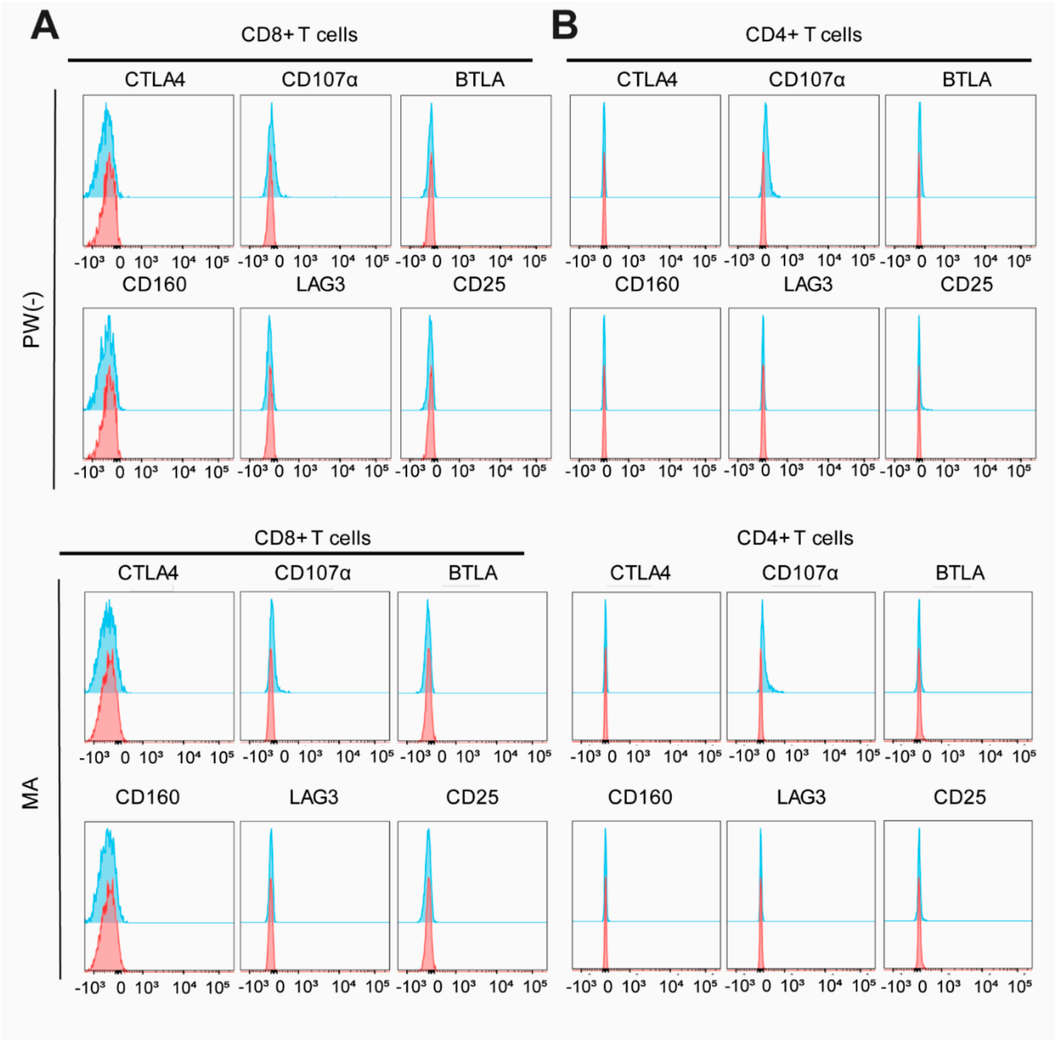

Supplemental Figure S1. CD8+ and CD4+ T cell immune marker expression of ex vivo cells from patients with GCPM. Fresh ex vivo cells from peritoneal washing (PW(-)) or malignant ascites (MA) were stained with anti-CD45, anti-CD3, anti-CD4, and anti-CD8 antibodies and the markers shown in the figure. CD8+ T cells (A) and CD4+ T cells (B) were gated and analyzed as a histogram for immune markers. One representative of each marker is shown in the figure.

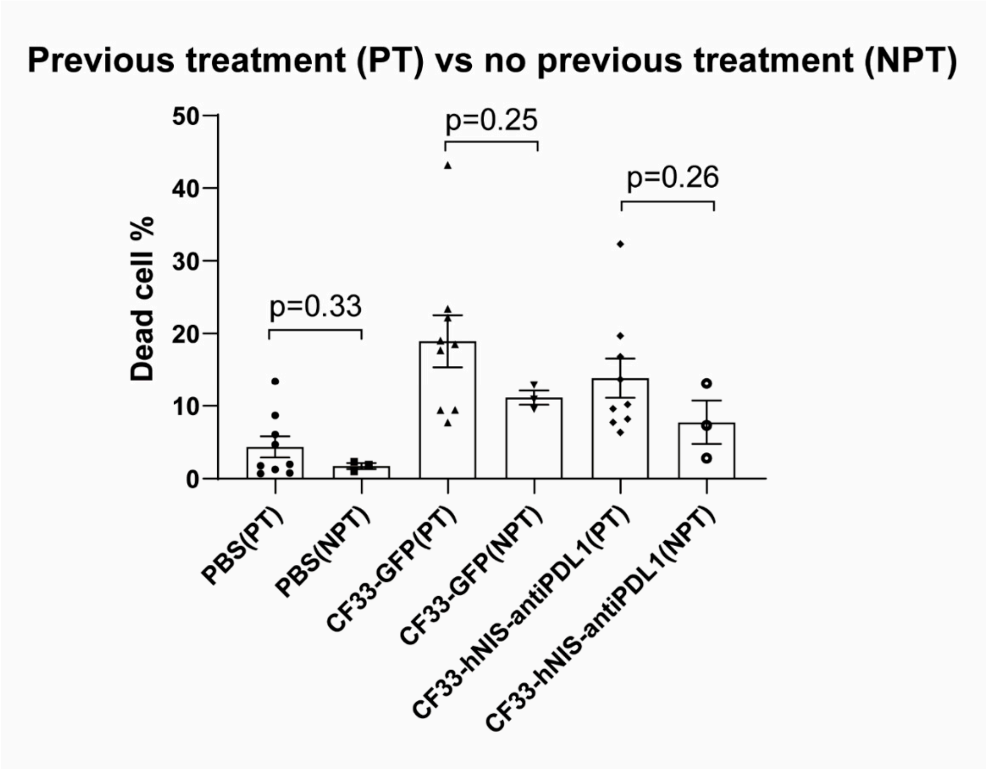

Supplemental Figure S2. Evaluation of previous treatment versus no previous treatment for the efficacy of virus-killing ex vivo cancer cells at the 15 hour time point. A total of 12 samples, of which there were 9 samples with previous treatment (PT) and 3 samples with no previous treatment (NPT) before specimen collection, were treated with PBS, CF33-GFP (MOI=3), or CF33-hNIS-antiPDL1 (MOI=3) for 15 hours and the dead cell percentage of each group is shown in the figure (Mean±SEM).
